# Validation and factorial invariance of the breastfeeding self-efficacy scale-short form in Ecuadorian mothers

**DOI:** 10.3389/fgwh.2026.1707796

**Published:** 2026-01-30

**Authors:** Ana Lizette Rojas-Rodríguez, Víctor López-Guerra, Cristian Trelles-Guarnizo, Paulo Rodríguez-Romero

**Affiliations:** 1 Department of Health Sciences, Universidad Técnica Particular de Loja, Loja, Ecuador; 2 Department of Psychology, Universidad Técnica Particular de Loja, Loja, Ecuador

**Keywords:** breastfeeding self-efficacy, Ecuador, factorial invariance, maternal breastfeeding, mothers, psychometric analysis, scale

## Abstract

**Background:**

Breastfeeding is one of the most cost-effective public health interventions to improve child survival and development. A key determinant of breastfeeding initiation and continuation is maternal self-efficacy, defined as a mother's confidence in her ability to breastfeed. The Breastfeeding Self-Efficacy Scale–Short Form (BSES-SF) is one of the most widely used instruments internationally to assess this construct. However, no validation studies have been conducted in Ecuador, and evidence from Latin America remains limited, particularly regarding advanced psychometric analyses.

**Objective:**

To validate the Spanish version of the BSES-SF in Ecuadorian mothers with previous breastfeeding experience, examining its factorial structure, factorial invariance, reliability, and convergent validity.

**Methods:**

An instrumental psychometric study was conducted with 325 mothers recruited from public and private health centers in Loja, Ecuador, who had breastfeeding experience during the child's first two years of life. Confirmatory factor analysis (CFA) was used to compare competing factorial models, and multi-group CFA tested factorial invariance across maternal age groups. Reliability was assessed using Cronbach's alpha and McDonald's omega. Convergent validity was examined through correlations with psychological capital (PCQ-12) and positive mental health (PMH-9).

**Results:**

A second-order four-factor model showed the best fit to the data (*χ*²/df = 1.39; CFI = 0.998; TLI = 0.997; SRMR = 0.043; RMSEA = 0.035) and demonstrated full factorial invariance across age groups. Convergent validity was generally adequate, although one dimension showed average variance extracted values below the recommended threshold. The scale showed excellent overall reliability (*α* = 0.915; *ω* = 0.929) and significant positive correlations with psychological capital and positive mental health.

**Conclusion:**

The Spanish version of the BSES-SF is a valid and reliable instrument for assessing breastfeeding self-efficacy in Ecuadorian mothers. Clinically, it supports early identification of women at risk of low breastfeeding self-efficacy, facilitating timely and targeted breastfeeding support. From a public health and policy perspective, this psychometrically robust and culturally adapted instrument can inform breastfeeding promotion programs, equity-focused maternal health interventions, and monitoring of nutrition and health-related Sustainable Development Goals.

## 1 Introduction

Exclusive breastfeeding during the first six months of life, followed by its continuation alongside complementary feeding up to two years of age or beyond, constitutes one of the most effective and sustainable interventions for child health (1). Its universal practice could prevent more than 820,000 child deaths each year, mainly due to infections such as diarrhea and pneumonia, especially in low- and middle-income countries (2). In addition to reducing the risk of chronic non-communicable diseases, breastfeeding strengthens the mother–infant bond, promotes maternal psychological well-being, and supports the development of a balanced gut ecosystem, which is essential for healthy child growth (3–5). Complementarily, this practice contributes to environmental sustainability by reducing the carbon footprint associated with the production and use of industrial formulas (6, 7). Taken together, these benefits explain why breastfeeding has been recognized as a key strategy for achieving several Sustainable Development Goals, particularly those related to health, food security, and climate action.

Despite these well-documented benefits, breastfeeding rates remain suboptimal worldwide. Globally, only 46% of newborns are breastfed within the first hour of life, and between 44% and 48% of infants under six months of age receive exclusive breastfeeding, figures that remain below the 50% target established for 2025 (8). In Ecuador, 72.7% of children under two years of age were breastfed during the first postpartum hour, with a higher prevalence in rural areas (75.6%) than in urban areas (71.2%). Exclusive breastfeeding during the first six months reached 62.1% at the national level. However, relevant inequalities persist mothers with basic education show the highest rates (66.3%), whereas women with higher education present the lowest prevalence (50.2%) (9). These differences highlight the need for targeted interventions to reduce socio-educational gaps and promote equity in this essential practice for child health.

Understanding the complex interaction of factors that influence the decision to breastfeed and its duration is therefore essential. Among psychosocial determinants, self-efficacy conceptualized within Bandura's social cognitive theory as the belief in one's own ability to organize and execute actions required to achieve desired outcomes has been identified as a strong predictor of breastfeeding initiation and maintenance (10). According to Bandura's theoretical model, self-efficacy is constructed through four primary sources: mastery experiences, vicarious experiences, verbal persuasion, and physiological and affective states. Based on this theoretical framework, Dennis (11) developed the Breastfeeding Self-Efficacy Scale (BSES), a 33-item instrument designed to assess mothers' confidence in their ability to breastfeed, explicitly grounded in these four sources of self-efficacy. Although the scale demonstrated excellent psychometric properties, its length limited its applicability in large-scale studies and routine clinical settings. To address this limitation, Dennis subsequently proposed the Breastfeeding Self-Efficacy Scale–Short Form (BSES-SF), a 14-item abbreviated version that preserves the conceptual and psychometric integrity of the original instrument (12).

The Spanish adaptation conducted by Oliver-Roig (13) confirmed solid psychometric performance. Since then, the BSES-SF has been translated and validated in multiple cultural contexts including Chinese (14), Croatian (15), Italian (16), Swedish (17), Persian (18), Greek (19), German (20), Turkish (21), Malay (22), Singaporean (23), Slovak (24), Indian (25), United States populations (26), and Latin American populations such as Colombia (27), Chile (28), Mexico (29), and Brazil (30) with reliability coefficients ranging between 0.81 and 0.94. Nevertheless, studies have reported inconsistent factorial structures: some confirmed a unidimensional model (15–18, 31, 32), while others supported bifactorial solutions (19, 23, 27, 28, 33) or even four-factor structures (24).

Despite its widespread use, most validations present methodological limitations: they are mainly based on exploratory analyses, omit rigorous confirmatory testing comparing alternative models, do not assess factorial invariance across subgroups, and rarely report McDonald's omega. To date, no study in Latin America has applied these advanced procedures or evaluated the factorial invariance of the BSES-SF.

In addition, although previous studies have evaluated the convergent validity of the BSES-SF with psychological constructs such as self-esteem, stress management, perceived social support, quality of life, and symptoms of anxiety and depression, the relationship between breastfeeding self-efficacy and positive mental health resources, such as psychological capital, remains unexplored (22, 34). Psychological capital, defined as a higher-order construct comprising general self-efficacy, hope, optimism, and resilience, has been shown to be a protective factor against various forms of psychological distress. Exploring its association with breastfeeding self-efficacy could provide new perspectives for the design of interventions that promote maternal psychological well-being and, consequently, breastfeeding continuation.

This gap is critical, as the assessment of factorial invariance ensures that the instrument measures breastfeeding self-efficacy equivalently across sociodemographic subgroups, while McDonald's omega provides a more accurate estimate of reliability than Cronbach's alpha, particularly in multidimensional structures. Specifically, testing factorial invariance across maternal age groups allows examination of whether the BSES-SF maintains the same measurement structure, factor loadings, and item functioning among younger and older mothers, ensuring that observed differences in self-efficacy reflect true variations rather than age-related measurement bias.

In this context, a critical research gap persists in Latin America, particularly in Ecuador, where no psychometric validation of the BSES-SF has been conducted to date. Moreover, existing studies in the region have not examined measurement equivalence across key sociodemographic subgroups, limiting the interpretability and comparability of breastfeeding self-efficacy scores. Addressing this gap, the present study represents the first validation of the BSES-SF in Ecuador and the first in Latin America to formally test factorial invariance. By applying confirmatory factor analysis, measurement invariance procedures, and advanced reliability estimation, this study provides robust and culturally relevant psychometric evidence to support equitable assessment, cross-group comparisons, and evidence-informed maternal health interventions in the region.

### 1.1 Research hypotheses

1. The Spanish version of the BSES-SF will present either a unidimensional or a bifactorial structure with adequate fit indices in confirmatory factor analysis, replicating previous findings in Spanish-speaking contexts.
2. The factorial structure of the BSES-SF will demonstrate factorial invariance across maternal age groups, indicating that the construct of breastfeeding self-efficacy is assessed equivalently among younger and older mothers.
3. The scale will show high internal consistency, with Cronbach's *α* ≥ 0.80 and McDonald's *ω* ≥ 0.80, supporting its reliability as an assessment instrument.
4. Positive and statistically significant correlations will be observed between breastfeeding self-efficacy and the dimensions of psychological capital (general self-efficacy, hope, optimism, resilience), as well as with indicators of positive mental health, supporting its convergent validity.

## 2 Materials and methods

### 2.1 Study type and research design

An instrumental study with a psychometric approach was conducted, with the primary objective of validating an assessment instrument in a sample of postpartum women. This type of design is appropriate for analyzing the psychometric properties of a scale, including its factorial structure, internal consistency, and validity, and is fundamental to ensuring the quality of measurements in specific contexts (35).

### 2.2 Participants

The study population consisted of mothers aged 18 years and older, with previous breastfeeding experience and with children within the first two years of life, who attended three healthcare facilities in southern Ecuador: a public primary-level health center, a secondary-level hospital, and a private tertiary-level institution providing specialized healthcare services, all located in the city of Loja, Ecuador. Data collection was conducted between October 7, 2024, and March 28, 2025.

The final sample included 325 mothers aged 18 years and older (≥18 years), selected through non-probabilistic convenience sampling. Maternal age was defined at the time of data collection. Consequently, although some participants may have initiated breastfeeding during adolescence, all were adults at the time of study inclusion. The minimum age criterion was established in accordance with national ethical regulations and international research standards that allow autonomous informed consent only in adult populations.

The minimum required sample size was initially estimated using a standard formula for proportions commonly applied in cross-sectional studies, assuming maximum variability (*p* = 0.50), a 95% confidence level (Z = 1.96), and a margin of error of 5%. Based on these parameters, a minimum sample size of approximately 300 participants was required to ensure adequate precision of the estimates. This conservative approach was adopted to account for potential non-response and incomplete questionnaires.In addition, sample size adequacy was evaluated following methodological recommendations for confirmatory factor analysis (CFA). Specifically, the achieved sample (*N* = 325) exceeded the commonly accepted threshold of 10–20 participants per item, supporting sufficient statistical power for model estimation and parameter stability (36).

Furthermore, this sample size is considered appropriate for the use of the Weighted Least Squares Mean and Variance adjusted (WLSMV) estimator, which is recommended for ordinal data in psychometric validation studies (37).

Although non-probabilistic sampling was employed, an *a priori* sample size estimation was conducted to ensure adequate precision and confidence. The achieved sample of 325 mothers therefore satisfies both theoretical and empirical standards for CFA and structural modeling with ordinal indicators. Efforts were made to include participants from different socioeconomic backgrounds to reduce potential bias related to socioeconomic status, age, and perinatal history.

The mean age of participants was 28.55 years (SD = 6.71; range = 18–48 years). Inclusion criteria were: (1) being 18 years of age or older at the time of data collection; (2) having previous breastfeeding experience with a child within the first two years of life; and (3) providing voluntary informed consent and completing all items of the applied instruments.

Table 1 presents the sociodemographic characteristics of the participants. Most mothers had completed secondary education (51.4%) and belonged to the middle socioeconomic class (79.7%). Regarding marital status, 38.8% were single and 35.7% were married. Vaginal delivery was reported by 56.3% of participants, and 60.3% indicated previous breastfeeding experience. Additionally, 80.6% reported having received information about breastfeeding. Most participants lived in nuclear families (66.8%) and resided in urban areas (95%).

**Table 1:** Sociodemographic characteristics of the study participants.

Variable	N	%	M	SD/R
Age			28.55	SD = 6.71.
				18–48
Education level				
Primary	8	2.5		
Incomplete primary	32	9.8		
Secondary	167	51.4		
Undergraduate	96	29.5		
Postgraduate	22	6.8		
Economic status				
Low	36	11.1		
Middle	279	79.7		
High	10	2.9		
Marital status				
Single	126	38.8		
Married	116	35.7		
Divorced	7	2.2		
Cohabiting	76	23.4		
Delivery method				
Vaginal	183	56.3		
Cesarean	141	43.4		
Number of living children				
One	147	45.2		
Two	103	31.7		
Three or more	75	23.1		
Previous breastfeeding experience				
Yes	196	60.3		
No	128	39.4		
Breastfeeding information				
Yes	262	80.6		
No	63	19.4		
Family structure				
Nuclear	217	66.8		
Extended	83	25.5		
Single parent	21	6.5		
Residence				
Urban	309	95.1		
Rural	16	4.9		

N, number; M, mean; SD, standard deviation; R, range.

The sociodemographic characteristics of the sample are detailed in Table 1.

### 2.3 Instruments

Four instruments were used in this study. The primary tool was the Breastfeeding Self-Efficacy Scale–Short Form (BSES-SF), whose validity and reliability analysis constituted the central objective of the research. To evaluate its convergent validity, two complementary scales were applied: the Psychological Capital Questionnaire (PCQ-12) and the Positive Mental Health Scale (PMH-9). In addition, a sociodemographic questionnaire was used to characterize the participants and contextualize the statistical analyses.

*Sociodemographic questionnaire*. This form was designed to collect basic information about the participants, including age, educational level, marital status, number of children, previous breastfeeding experience, type of delivery, access to breastfeeding information, family structure, and geographic origin.

*Breastfeeding Self-Efficacy Scale–Short Form (BSES-SF)*. Breastfeeding Self-Efficacy Scale–Short Form (BSES-SF).

Breastfeeding self-efficacy was assessed using the abbreviated version of the Breastfeeding Self-Efficacy Scale–Short Form (BSES-SF), originally developed by Dennis (11) and using the Spanish version previously translated and validated by Oliver-Roig et al. (13). This version consists of 14 items that assess mothers' perceived confidence in their ability to breastfeed, including breastfeeding techniques, coping with difficulties, personal confidence, and perceived support. Items are rated on a 5-point Likert scale (1 = strongly disagree to 5 = strongly agree), with higher scores indicating greater breastfeeding self-efficacy. The Spanish version by Oliver-Roig et al. has demonstrated excellent psychometric properties, including high internal consistency (Cronbach's *α* = 0.92), a unidimensional factorial structure, and adequate convergent validity.

In the present study, the Spanish version developed by Oliver-Roig et al. was used without modification as the linguistic base instrument. No new direct or back-translation process was undertaken. Instead, the methodological focus was placed on verifying the semantic clarity and contextual appropriateness of the existing Spanish items for use among Ecuadorian mothers.

*Psychological Capital Questionnaire (PCQ-12)*. Psychological capital was assessed using the 12-item short version of the Psychological Capital Questionnaire (PCQ-12) (38) Lorenz et al., 2022). This instrument conceptualizes psychological capital as a higher-order construct comprising four dimensions: hope (items 1–3), optimism (items 4–6), resilience (items 7–9), and self-efficacy (items 10–12). Responses are provided on a six-point Likert scale ranging from strongly disagree to strongly agree, with higher scores indicating greater psychological capital. The Spanish adaptation of the PCQ-12, including the blind back-translation procedure, language validation, and pilot testing, was carried out by López-Guerra (39, 40), following established methodological guidelines (40, 41). In that study, the authors confirmed the factorial structure of the scale and reported excellent internal consistency for the total score (*α* = 0.93; *ω* = 0.93) and satisfactory reliability for its dimensions: hope (*α* = 0.80; *ω* = 0.80), optimism (*α* = 0.85; *ω* = 0.85), resilience (*α* = 0.79; *ω* = 0.81), and self-efficacy (*α* = 0.86; *ω* = 0.87).

*Positive Mental Health Scale (PMH-9).* Positive mental health was evaluated using the PMH-9, developed by Lukat (42) and adapted into Spanish by Boufellous (43). This unidimensional scale includes 9 items measuring emotional, psychological, and social well-being, rated on a 4-point Likert scale (0 = disagree to 3 = agree). In its validation with the general Spanish population, the PMH-9 showed excellent internal consistency (*α* = 0.96; *ω* = 0.97) and a unidimensional structure confirmed through confirmatory factor analysis. It also presented significant positive correlations with optimism (*r* = 0.79), resilience in the context of suicidal ideation (*r* = 0.92), general self-efficacy (*r* = 0.74), and perceived social support (*r* = 0.76), as well as negative correlations with anxiety (*r* = –0.80) and depression (*r* = –0.60). Given its brevity, cross-cultural validity, and psychometric robustness, this scale was considered appropriate to explore the convergent validity of the BSES-SF with indicators of positive mental health.

### 2.4 Procedure

The study was conducted in southern Ecuador between October 2024 and March 2025. Participants were approached in the waiting rooms of primary care consultations at public and private health centers in the city of Loja. Data collection was carried out through face-to-face surveys and digital forms (Google Forms), administered by the research team previously trained for this purpose. Before participation, the purpose of the study was explained in detail, and informed consent was obtained voluntarily. Participation was anonymous, confidential, and no financial compensation was offered. The study was approved by the Research Ethics Committee (code: 2024-03-INT-EO-RM-004) and conducted in accordance with the principles set forth in the Declaration of Helsinki (World Medical Association, 2013) (44).

Prior to the final administration of the questionnaire, a contextual verification process was conducted to ensure that the terminology and expressions used in the Spanish version of the BSES-SF developed by Oliver-Roig et al. were appropriate for the Ecuadorian sociocultural context. This process followed the International Test Commission (ITC) Guidelines for test adaptation (Second Edition) (37, 45), specifically those applicable when a previously translated and validated instrument is applied in a new cultural setting.

A formal content validity assessment was conducted using the Content Validity Index (CVI). A panel of five experts evaluated all items independently in terms of relevance, clarity, and cultural appropriateness for Ecuadorian mothers. The expert panel included a pediatrician, a breastfeeding specialist, a nutritionist with expertise in maternal–child health, a methodologist experienced in psychometric validation, and a public health researcher familiar with the local sociocultural context. Each item was rated on a four-point ordinal scale, and item-level CVI (I-CVI) and scale-level CVI (S-CVI) were calculated. The results showed I-CVI values ≥ 0.80 for all items and an overall S-CVI of 0.92, indicating adequate content validity. Based on these results, no items were modified or removed.

Subsequently, a pilot study was conducted with 30 Ecuadorian breastfeeding women to further evaluate item comprehension, semantic clarity, and cultural acceptability. The pilot testing was carried out in the same public and private healthcare facilities located in the city of Loja that were later included in the main study, ensuring contextual consistency between the pilot and the final data collection phases. The pilot participants presented sociodemographic and clinical characteristics comparable to those of the final study population.

Participants in the pilot phase reported clear understanding of all items and no difficulties in interpretation. Consistent with the expert panel review and the CVI results, the pilot study confirmed that the Spanish version of the BSES-SF was culturally appropriate and comprehensible for use among Ecuadorian mothers, and that no linguistic or cultural modifications to the scale were required.

No statistical analyses of reliability or factorial validity were conducted in the pilot sample, as this phase was designed exclusively for qualitative verification of clarity and cultural adequacy, in accordance with ITC recommendations. Psychometric analyses, including confirmatory factor analysis and reliability estimation, were therefore appropriately performed only in the full study sample (*N* = 325), which met established methodological criteria for such analyses. Data obtained during the pilot phase were used exclusively for this evaluative purpose and were not included in the final psychometric analyses.

This verification process ensured that the Spanish BSES-SF retained the original structure and conceptual meaning established by Oliver-Roig et al., while being suitable for application in the Ecuadorian context.

### 2.5 Data analysis

Statistical analyses were conducted using the open-source software JASP (version 0.95.0.0). First, the factorial structure of the instrument was assessed through a confirmatory factor analysis (CFA). Given that the scale uses a Likert-type format and therefore involves ordinal measurement, the estimation method applied was Weighted Least Squares Mean and Variance adjusted (WLSMV) with polychoric correlations. This estimator is considered the most appropriate for ordinal categorical data, as it produces robust parameter estimates and standard errors while providing accurate fit indices even when normality assumptions are violated. WLSMV is especially advantageous in large samples (*N* > 200) and has been widely recommended for psychometric research with ordinal response instruments, such as the BSES-SF, because it minimizes bias and improves the reliability of structural equation modeling results (46, 47).

For the analysis, six factorial models were estimated and compared in order to identify the structure that best fit the empirical data. Model fit was evaluated using the following indices: chi-square to degrees of freedom ratio (*χ*²/df), Bentler's Comparative Fit Index (CFI), Tucker–Lewis Index (TLI), Standardized Root Mean Square Residual (SRMR), and Root Mean Square Error of Approximation (RMSEA). The criteria used to determine model adequacy were: *χ*²/df ≤ 3 as acceptable and ≤2 as optimal (48). CFI and TLI ≥ 0.90 as acceptable and ≥0.95 as optimal; and RMSEA and SRMR ≤ 0.08 as acceptable and ≤0.05 as optimal (49).

Second, the Average Variance Extracted (AVE) was calculated to evaluate convergent validity, that is, to determine whether the items adequately represent the latent variable they are intended to measure rather than another construct. According to the acceptance criterion proposed by Fornell and Larcker (50), the AVE value should exceed 0.50, indicating that the construct explains more than 50% of the variance in its indicators, with the remainder attributed to measurement error.

Third, factorial invariance of the scale was tested as a function of maternal age. For this analysis, the sample was split into two groups based on the median, yielding groups of equivalent size. A multi-group confirmatory factor analysis (MG-CFA) was applied, considering the following levels of invariance: configural invariance (MC), which assesses the factorial structure without group restrictions; metric invariance (MM), which constrains factor loadings to equality across groups; scalar invariance (SC), which adds equality constraints on factor loadings and intercepts; and strict invariance (ST), which also includes equality of measurement residuals. Invariance levels were evaluated according to Cheung et al. (2002), with acceptance criteria of a change in CFI (ΔCFI) ≤ 0.01 and a change in RMSEA (ΔRMSEA) ≤ 0.015.

Fourth, the reliability of the Breastfeeding Self-Efficacy Scale–Short Form (BSES-SF) was analyzed through internal consistency, using both Cronbach's alpha (*α*) and McDonald's omega (*ω*). Values ≥ 0.70 for both coefficients were considered indicative of satisfactory reliability (47, 48). McDonald's omega was prioritized given its robustness for ordinal response scales such as the BSES-SF, as it does not depend on the number of items and, by relying on factor loadings, provides more stable estimates. Furthermore, omega is particularly suitable in contexts where the assumption of tau-equivalence is not met, which is frequent in practice (51, 52).

Fifth, convergent validity was examined by analyzing the correlations between the BSES-SF and two theoretically related constructs: the Psychological Capital Scale (PCQ-12) and the Positive Mental Health Scale (PMH-9). Significant positive correlations were expected, supporting the hypothesis that higher levels of breastfeeding self-efficacy are associated with greater psychological resources and better indicators of positive mental health. Importantly, this analysis represents an innovation of the present study, since previous validations of the BSES-SF have rarely examined its convergence with constructs of positive mental health and psychological capital.

Finally, a descriptive analysis of participants' responses to the BSES-SF was carried out, reporting the mean (M) and standard deviation (SD), based on the factorial structure that showed the best fit to the data.

## 3 Results

### 3.1 Confirmatory factor analysis

A confirmatory factor analysis (CFA) was conducted to evaluate the internal structure of the Breastfeeding Self-Efficacy Scale–Short Form (BSES-SF). Based on previous literature, five competing theoretical models were tested in order to identify the factorial structure that provided the best empirical fit.

**Model 1:** A unidimensional structure originally proposed by Dennis (34) and supported in several subsequent validations: Gregory (53); McCarter (26); Dodt (30); Oliver-Roig (13); Amini (18); Gerhrdsson (17); Sandhi (31); Chipojola (22).

**Model 2:** A two-factor solution distinguishing intrapersonal thoughts (items 1–6) and breastfeeding techniques (items 7–14), as reported in Chile Andrade (28); and Colombia, Trujillo (27). Comparable bifactorial distinctions have been described with alternative labels, such as cognitive–emotional vs. breastfeeding techniques by Economou (19) and Yang (34).

**Model 3:** The two-factor model of De Roza (23), comprising cognitive aspects (items 1–8) and breastfeeding management (items 9–14).

**Model 4:** A four-factor solution proposed by Mazúchová (24), consisting of Intrapersonal Thoughts (items 1–4), Coping and Support (items 5–7), and Practical Aspects of Breastfeeding (items 8–10) and Breastfeeding Technique (items 11–14).

**Model 5:** A second-order model incorporating the four dimensions identified by Mazúchová (24).

**Figure 1:**
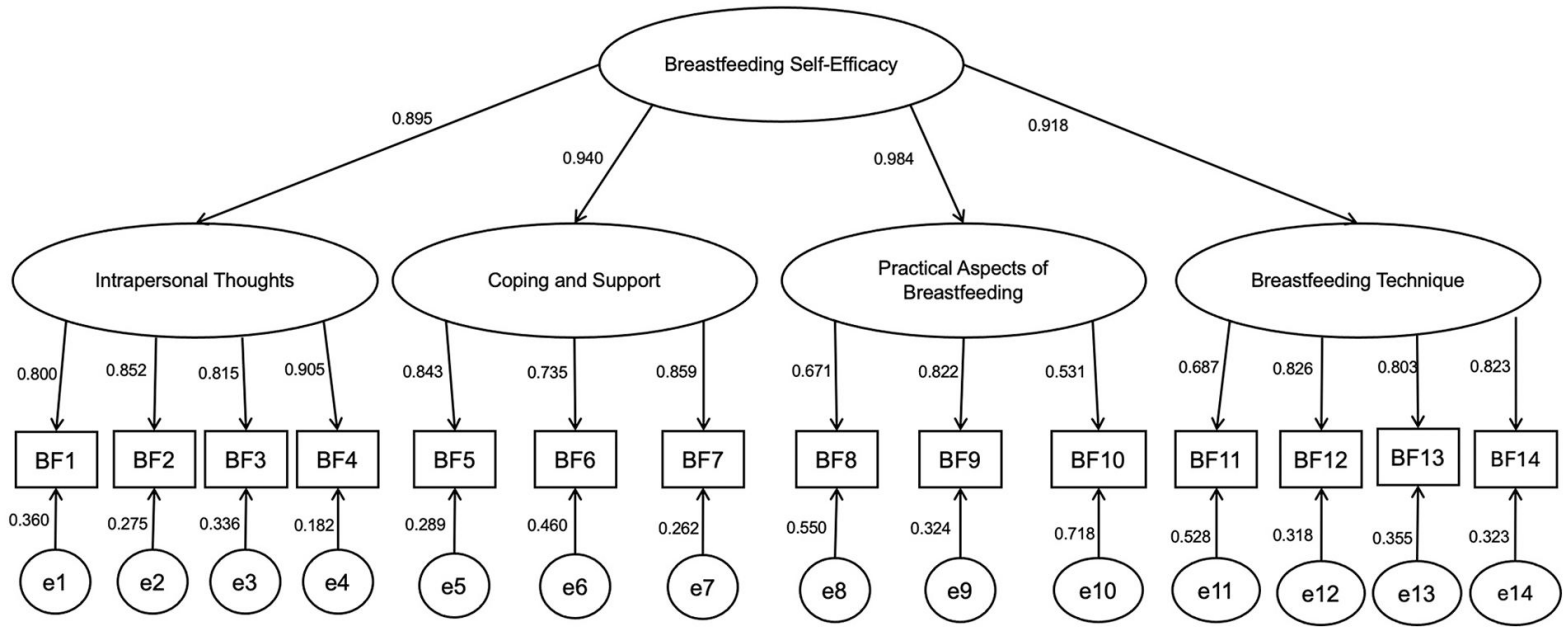
Diagram of the second-order confirmatory factor analysis model of the breastfeeding self-efficacy scale–short form (BSES-SF), with four first-order factors: intrapersonal thoughts, coping and support, practical aspects of breastfeeding, and breastfeeding technique.

Based on the fit indices, Model 5 a second-order model with four first-order factors demonstrated the best empirical fit to the data (*χ*²/df = 1.39; CFI = 0.998; TLI = 0.997; SRMR = 0.043; RMSEA = 0.035). This structure conceptualizes breastfeeding self-efficacy as a higher-order construct composed of four dimensions: Breastfeeding Technique (items 11–14), Intrapersonal Thoughts (items 1–4), Coping and Support (items 5–7), and Practical Aspects of Breastfeeding (items 8–10). As shown in Table 2 and Figure 1, this model provided the most parsimonious and theoretically coherent representation of the internal structure of the BSES-SF in this sample of Ecuadorian mothers.

**Table 2:** Fit indices of the models used in the confirmatory factor analysis.

MODEL	*X^2^/df*	CFI	TLI	SRMS	RMSEA
Model 1	3.90	0.951	0.949	0.057	0.095
Model 2	3.02	0.966	0.959	0.048	0.074
Model 3	3.37	0.960	0.952	0.053	0.085
Model 4	2.49	0.976	0.970	0.042	0.068
Model 5	1.39	0.998	0.997	0.043	0.035

*χ*²/df, Chi-square divided by degrees of freedom; CFI, Bentler's comparative fit index; TLI, Tucker–Lewis index; SRMR, standardized root mean square residual; RMSEA, root mean square error of approximation.

It is noteworthy that Model 4, the four-factor solution originally reported, also exhibited excellent fit indices (24). However, the correlations between its factors were excessively high (ranging from 0.814 to 0.940), suggesting redundancy among dimensions. This motivated the specification of the second-order structure, which not only preserved the multidimensionality of the instrument but also accounted for the strong interrelationships among factors under a unified higher-order construct. This result led to the specification of the second-order structure to account for the observed inter-factor correlations.

### 3.2 Convergent validity

Convergent validity was examined through the Average Variance Extracted (AVE) for each factor in the four-factor second-order model proposed by Mazúchová (24) consisting of Intrapersonal Thoughts (items 1–4), Coping and Support (items 5–7), Practical Aspects of Breastfeeding (items 8–10), and Breastfeeding Technique (items 11–14).

The results indicated that Intrapersonal Thoughts (F1; AVE = 0.712), Coping and Support (F2; AVE = 0.663), and Breastfeeding Technique (F4; AVE = 0.619) exceeded the recommended cut-off value of 0.50 thus demonstrating strong convergent validity. In contrast, Practical Aspects of Breastfeeding (F3; AVE = 0.469) did not reach the threshold, suggesting limited convergent validity for this dimension (49). This may be related to the small number of items (items 8–10) and their relatively higher residual variance.

### 3.3 Factorial invariance

The multi-group confirmatory factor analysis (MG-CFA) supported the factorial invariance of the BSES-SF second-order structure with four first-order dimensions. The baseline configural model showed excellent fit indices [*χ*² = 165.02, df = 138, CFI = 0.998, SRMR = 0.056, RMSEA = 0.035, 90% CI (0.000, 0.053)], confirming that the same second-order four-factor structure was replicated across maternal age groups.

When equality constraints were imposed on factor loadings (metric invariance), model fit remained satisfactory (*χ*² = 175.91, df = 180, CFI = 1.000, *Δ*CFI = 0.002, RMSEA = 0.000), indicating that the contribution of the items to the latent dimensions was equivalent across younger (≤28 years) and older (≥29 years) mothers. As shown Table 3.

**Table 3:** Factorial invariance of the SWLS for the total sample and by age.

Model	*χ* ^2^	df	C-M	Δχ^2^	Δ*df*	CFI	ΔCFI	RMR	RMSEA (CI 90%)	ΔRMSEA
Entire Group	96.08	69	-	-	-	0.998	-	0.043	0.035 (0.015, 0.050)	-
≤28	83.23	69	-	-	-	0.998	-	0.055	0.035 (0.000, 0.059)	-
≥29	81.79	69	-	-	-	0.998	-	0.057	0.035 (0.000, 0.062)	-
MC	165.02	138	-	-	-	0.998	-	0.056	0.035 (0.000, 0.053)	-
MM	175.91	180	MM–MC	−10.89	−42	1.000	0.002	0.056	0.000 (0.000, 0.033)	0.035
SC	207.5	193	SC–MM	−31.59	13	0.999	0.001	0.057	0.022 (0.000,.041)	0.022
ST	207.5	193	ST–SC	0	0	0.999	0	0.057	0.022 (0.000,.0041)	0

χ², Chi-square; df, degrees of freedom; C-M, comparison of factorial invariance models; Δχ², change in chi-square; Δdf, change in degrees of freedom; CFI, comparative fit index; ΔCFI, change in CFI; SRMR, standardized root mean square residual; RMSEA, root mean square error of approximation; CI, confidence interval; MC, configural model; MM, metric model; SC, scalar model; ST, strict model.

The scalar invariance model, which constrained both loadings and intercepts, also demonstrated good fit (*χ*² = 207.50, df = 193, CFI = 0.999, ΔCFI = 0.001, RMSEA = 0.022). Finally, strict invariance—imposing equality on residual variances—was equally supported (*χ*² = 207.50, df = 193, CFI = 0.999, ΔCFI = 0, RMSEA = 0.022).

### 3.4 Internal consistency

The internal consistency of the BSES-SF, conceptualized as a second-order construct with four first-order dimensions, was assessed using both Cronbach's alpha (*α*) and McDonald's omega (*ω*). These complementary indicators provide a robust evaluation of reliability, particularly in multidimensional instruments.

The total scale demonstrated excellent reliability (*α* = 0.915; *ω* = 0.929), exceeding the recommended threshold of 0.80 and confirming that the items consistently measure the overarching construct of breastfeeding self-efficacy with strong internal homogeneity. At the factor level, Intrapersonal Thoughts (F1) showed high reliability (*α* = 0.869; *ω* = 0.846), while Coping and Support (F2) also demonstrated adequate consistency (*α* = 0.789; *ω* = 0.780). Practical Aspects of Breastfeeding (F3) presented lower coefficients (*α* = 0.580; *ω* = 0.612), suggesting the need for further refinement of these items to improve internal coherence. Finally, Breastfeeding Technique (F4) exhibited satisfactory reliability (*α* = 0.791; *ω* = 0.795).

### 3.5 Convergent validity with related constructs

Convergent validity of the BSES-SF was examined through correlations with the Psychological Capital Questionnaire (PsyCap), its four dimensions (hope, optimism, resilience, and self-efficacy), and the Positive Mental Health Scale. As shown in Table 4, all correlations were positive and statistically significant (*p* < 0.001), supporting the adequacy of the measure.

**Table 4:** Correlation matrix between the BSES-SF and other measures related to positive mental health.

Variables	Total Psychological Capital	Hope	Optimism	Resilience	Self-efficacy	Positive Mental Health
Breastfeeding Self-Efficacy Scale (BSES-SF)	0.538*	0.511*	0.530*	0.366*	0.532*	0.530*
Factor 1: Intrapersonal Thoughts	0.453*	0.405*	0.444*	0.283*	0.472*	0.449*
Factor 2: Coping and Support	0.463*	0.467*	0.477*	0.325*	0.417*	0.438*
Factor 3: Practical Aspects of Breastfeeding	0.468*	0.448*	0.462*	0.341*	0.441*	0.448*
Factor 4: Breastfeeding Technique	0.512*	0.501*	0.469*	0.353*	0.544*	0.526*

* *p* < 0.001.

Based on Cohen guidelines (54) the total BSES-SF score demonstrated strong associations with overall psychological capital (*r* = 0.538), hope (*r* = 0.511), optimism (*r* = 0.530), self-efficacy (*r* = 0.532), and positive mental health (*r* = 0.530). The correlation with resilience was moderate (*r* = 0.366), though still significant.

At the factor level, Intrapersonal Thoughts, Coping and Support, Practical Aspects of Breastfeeding, and Breastfeeding Technique all showed consistent positive correlations with psychological resources, ranging from moderate to strong (*r* = 0.283–0.544). The Breastfeeding Technique factor showed the highest correlations across constructs.

### 3.6 Descriptive analysis

Participating mothers (*N* = 325) demonstrated consistently high levels of breastfeeding self-efficacy across the four dimensions of the BSES-SF. The mean scores for each factor were: Intrapersonal Thoughts (M = 4.44, SD = 0.745), Coping and Support (M = 4.42, SD = 0.704), Practical Aspects of Breastfeeding (M = 4.37, SD = 0.681), and Breastfeeding Technique (M = 4.51, SD = 0.606). These values, all close to the maximum of the scale, indicate favorable perceptions in both the cognitive–emotional and technical–practical domains of breastfeeding.

**Figure 2:**
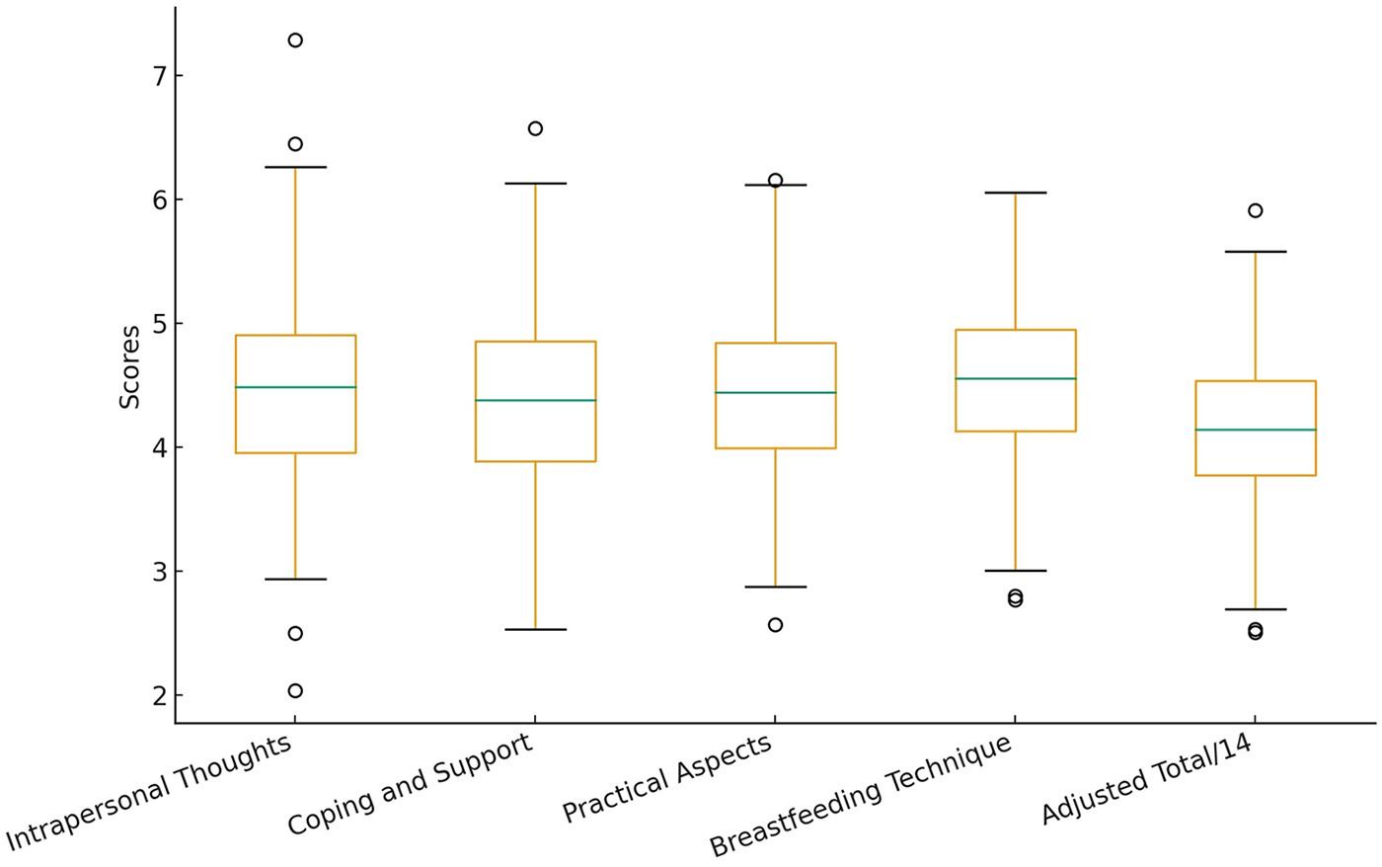
The boxplot below illustrates the distribution of the BSES-SF adjusted total score (per item) and the four dimensions based on simulated data using reported means and standard deviations. This representation allows visualizing the variability and clustering around the upper range of the scale.

Regarding the total score, mothers obtained an average of 57.9 points (SD = 7.80; range = 13.1–65.4). When divided by the 14 items of the scale, this yields a mean score per item of 4.14, placing participants in the upper range of the self-efficacy continuum. The median score was 59.4. As show Figure 2.

## 4 Discussion

The main objective of this study was to evaluate the psychometric properties and factorial structure of the Spanish short version of the Breastfeeding Self-Efficacy Scale (BSES-SF) in a sample of Ecuadorian mothers. This work addresses the absence of validated instruments in Ecuador to reliably assess maternal breastfeeding self-efficacy, a construct recognized as a key determinant in the initiation and continuation of breastfeeding (11, 12). By providing a culturally adapted and psychometrically robust tool, this study contributes to strengthening maternal and child health promotion strategies in the Latin American context.

Importantly, the findings are valid for mothers with recent breastfeeding experience (0–24 months of the child's life) and complement the literature that has assessed the BSES-SF across different perinatal periods, including prenatal and immediate postpartum stages (13, 24, 27, 28). Evaluating self-efficacy beyond the immediate puerperium is particularly relevant, as the construct reflects perceived capabilities that are continuously shaped by accumulated maternal experience. The second-order factorial structure, the invariance across maternal age groups, and the convergent validity observed in this study support the stability of the construct within this temporal framework.

### 4.1 Confirmatory factor analysis

The confirmatory factor analysis provided robust support for a second-order model of the BSES-SF in Ecuadorian mothers. Among the five competing alternatives, the hierarchical model (Model 5), comprising four first-order factors (Intrapersonal Thoughts, Coping and Support, Practical Aspects of Breastfeeding, and Breastfeeding Technique), showed the best empirical fit, with excellent indices across all parameters (*χ*²/df = 1.39; CFI = 0.998; TLI = 0.997; SRMR = 0.043; RMSEA = 0.035). This factorial solution conceptualizes breastfeeding self-efficacy as a higher-order construct that integrates multiple but strongly interrelated dimensions.

These findings are theoretically consistent with Bandura's social cognitive theory, which defines self-efficacy as individuals' beliefs in their capabilities to organize and execute the actions required to manage prospective situations (10). In this context, the hierarchical model illustrates how mothers' confidence in breastfeeding emerges from the interaction of cognitive, emotional, behavioral, and social sources of efficacy. Intrapersonal Thoughts and emotional regulation align with affective and cognitive sources of efficacy; Coping and Support reflects the influence of social persuasion; and Breastfeeding Technique and Practical Aspects correspond to mastery experiences, identified by Bandura as the most powerful determinant of efficacy beliefs (10).

Although the four-factor solution (Model 4) originally reported also demonstrated good fit indices, the excessively high inter-factor correlations (0.814–0.940) indicated redundancy among dimensions (24). Modeling these dimensions as first-order factors subsumed under a second-order construct provided a more parsimonious and theoretically coherent framework, consistent with Bandura's proposition that self-efficacy arises from multiple interacting determinants rather than isolated domains (10).

Unlike most previous validations in Latin America and other regions, which relied mainly on exploratory factor analysis (27, 28), the present study applied a confirmatory factor analytic approach. The CFA allows direct testing of theoretically derived models, provides rigorous evaluation of model fit, and reduces the risk of overfitting or misinterpretation. This methodological strength reinforces the psychometric validity of the BSES-SF in the Ecuadorian context and supports the use of confirmatory strategies in cross-cultural maternal health research.

### 4.2 Convergent validity

The convergent validity analysis of the four-factor second-order model provided additional evidence for the construct validity of the BSES-SF. Three of the four dimensions: Intrapersonal Thoughts (AVE = 0.712), Coping and Support (AVE = 0.663), and Breastfeeding Technique (AVE = 0.619) exceeded the recommended threshold of 0.50 (49), confirming that these latent factors adequately capture the variance explained by their indicators. This pattern is consistent with Bandura's view of self-efficacy as a multidimensional belief system integrating affective, cognitive, and behavioral information (10).

In contrast, the Practical Aspects of Breastfeeding dimension (AVE = 0.469) did not reach the cut-off value, suggesting weaker convergent validity. This limitation may be related to the small number of items and their higher residual variance, which can reduce internal consistency. Similar findings have been reported in other cross-cultural validations of the BSES-SF, particularly in shorter subscales (55). Overall, these results support the adequacy of the hierarchical model while highlighting areas for future refinement.

### 4.3 Factorial invariance

The present study provides strong evidence for the factorial invariance of the BSES-SF across maternal age groups. The multi-group confirmatory factor analysis supported configural, metric, scalar, and strict invariance, indicating that breastfeeding self-efficacy is measured equivalently in younger and older mothers. This ensures that observed group differences reflect true variation in the construct rather than measurement bias.

From a theoretical perspective, this finding aligns with Bandura's conceptualization of self-efficacy as a coherent psychological mechanism that operates consistently across developmental stages while remaining sensitive to experience and context (10). Methodologically, this represents an important contribution, as most previous validations of the BSES-SF have not examined measurement invariance across demographic subgroups (12).

### 4.4 Internal consistency

The BSES-SF demonstrated excellent internal consistency at the total scale level (*α* = 0.915; *ω* = 0.929), exceeding recommended thresholds. Three of the four dimensions also showed satisfactory reliability coefficients, while the Practical Aspects of Breastfeeding dimension exhibited lower values (*α* = 0.580; *ω* = 0.612). This pattern is consistent with the systematic review by Dennis (12), which reported greater variability in reliability estimates among non-English adaptations.

The lower reliability observed in this dimension may reflect contextual and cultural differences in how practical breastfeeding behaviors are perceived and experienced. Bandura emphasized that self-efficacy beliefs are shaped by sociocultural and environmental contingencies, which may differentially influence specific domains of efficacy across settings (10).

### 4.5 Convergent validity with related constructs

Positive and statistically significant correlations between breastfeeding self-efficacy, psychological capital, and positive mental health provide strong evidence of convergent validity. These findings are consistent with Bandura's assertion that self-efficacy plays a central role in motivation, emotional regulation, and adaptive behavior (10). Mothers with higher breastfeeding self-efficacy also reported greater hope, optimism, resilience, and general self-efficacy, situating the construct within a broader framework of positive psychological resources.

Previous studies have primarily focused on associations between the BSES-SF and negative outcomes such as postpartum depression (12). In contrast, the present study expands the evidence base by demonstrating associations with promotive psychological constructs, consistent with Bandura's emphasis on self-efficacy as a driver of positive functioning rather than merely a buffer against risk.

### 4.6 Descriptive analysis

The mean BSES-SF score observed in this sample (57.9 ± 7.8) indicates a high level of perceived breastfeeding self-efficacy, comparable to values reported in European populations (56) and higher than those documented in several Asian contexts (18, 32). These differences suggest that cultural norms, institutional support, and social environments may influence efficacy beliefs, in line with Bandura's concept of reciprocal determinism between individual, behavior, and environment (Bandura, 1986).

### 4.7 Limitations

Several limitations should be acknowledged. First, although the sample was obtained through a non-probabilistic convenience strategy and heterogeneity across sociodemographic variables was ensured, generalizability remains limited. Future studies should adopt probabilistic and multicenter designs.

Second, the cross-sectional design does not allow assessment of test–retest reliability or predictive validity. Longitudinal studies are needed to examine temporal stability and predictive capacity. Although predictive validity was not examined in this study, previous research has shown that lower BSES-SF scores predict earlier breastfeeding cessation (12).

Third, convergent validity was assessed, but discriminant and predictive validity were not evaluated. Finally, as with all self-report measures, response bias cannot be fully excluded, although standardized administration procedures and recent breastfeeding experience may have mitigated this limitation.

### 4.8 Implications for practice, research, and policy

The validated BSES-SF provides health professionals with a reliable and theoretically grounded instrument to identify mothers at risk of breastfeeding discontinuation and to guide targeted interventions. Within the Andean and broader Latin American context characterized by marked sociocultural, educational, and health-system heterogeneity this tool enables a more nuanced assessment of maternal breastfeeding self-efficacy and supports equity-oriented approaches to maternal health. Consistent with Bandura's social cognitive framework, effective interventions should focus on strengthening mastery experiences, facilitating vicarious learning, enhancing culturally responsive social support, and addressing emotional and contextual barriers that may undermine breastfeeding confidence (10).

From a research perspective, this validation contributes novel psychometric evidence from an underrepresented region and enables meaningful cross-cultural comparisons of breastfeeding self-efficacy across Latin American countries and beyond. The demonstrated factorial invariance of the BSES-SF supports its use as a common metric for examining intercultural similarities and differences in maternal confidence, allowing researchers to disentangle true contextual effects from measurement bias.

From a policy standpoint, incorporating breastfeeding self-efficacy as a psychosocial indicator within maternal and child health systems may support monitoring progress toward Sustainable Development Goals 2 and 3 in settings facing persistent social inequities. By providing a culturally adapted and invariant measure, this study facilitates evidence-informed decision-making and the design of regionally relevant breastfeeding promotion strategies. Overall, integrating Bandura's theoretical model with rigorous psychometric validation underscores the value of linking psychological theory, measurement, and public health policy to advance maternal and child health in the Andean and Latin American context.

## 5 Conclusion

The findings of this study demonstrate that the Spanish version of the BSES-SF shows excellent validity and reliability indicators, confirming its suitability for assessing maternal breastfeeding self-efficacy in Ecuadorian mothers. This represents the first psychometric validation of the instrument conducted in Ecuador and in the Andean region, providing culturally adapted evidence that extends beyond the local context and may be applicable to other Spanish-speaking countries in Latin America.

The availability of a validated and invariant measure of breastfeeding self-efficacy has important implications for maternal health practice and research. In applied settings, the BSES-SF can be used to identify mothers with lower self-efficacy who may benefit from targeted and timely support, contributing to more equitable maternal health strategies by addressing psychosocial vulnerabilities that influence breastfeeding initiation, duration, and exclusivity. In this regard, the instrument supports the design of equity-oriented interventions aimed at reducing disparities in breastfeeding outcomes across different sociodemographic groups in Latin America.

From a public health and policy perspective, the validated BSES-SF provides a useful tool for informing and evaluating breastfeeding promotion programs aligned with the Sustainable Development Goals, particularly SDG 2 (Zero Hunger) and SDG 3 (Good Health and Well-being). By enabling the early identification of mothers at risk of breastfeeding discontinuation, the scale can support evidence-based decision-making and the implementation of timely interventions to strengthen breastfeeding practices within health systems.

Finally, the demonstrated factorial invariance of the BSES-SF allows for meaningful comparisons of breastfeeding self-efficacy across maternal age groups and supports its use in cross-national research. This facilitates comparative studies between countries and regions using a common, psychometrically sound metric, strengthening the evidence base for regional and international breastfeeding policies. Overall, the BSES-SF is consolidated as a strategic resource for research, clinical practice, and public health decision-making in maternal and child health across Latin America.

## Data Availability

The raw data supporting the conclusions of this article will be made available by the authors, without undue reservation.

## References

[1] Victora CG Bahl R Barros AJ França GV Horton S Krasevec J Breastfeeding in the 21st century: epidemiology, mechanisms, and lifelong effect. Lancet. (2016) 387(10017):475–90. 10.1016/S0140-6736(15)01024-7 26869575

[2] North K Gao M Allen G Lee AC. Breastfeeding in a global context: epidemiology, impact, and future directions. Clin Ther. (2022) 44(2):228–44. 10.1016/j.clinthera.2021.11.017 34973827

[3] Vandenplas Y Berger B Carnielli VP Ksiazyk J Lagström H Sanchez Luna M Human milk oligosaccharides: 2′-fucosyllactose (2′-FL) and lacto-N-neotetraose (LNnT) in infant formula. Nutrients. (2018) 10(9):1161. 10.3390/nu10091161 30149573 PMC6164445

[4] Raspini B Vacca M Porri D De Giuseppe R Calabrese FM Chieppa M Early life microbiota colonization at six months of age: a transitional time point. Front Cell Infect Microbiol. (2021) 11:590202. 10.3389/fcimb.2021.590202 33842380 PMC8032992

[5] Carr LE Virmani MD Rosa F Munblit D Matazel KS Elolimy AA Role of human milk bioactives on infants’ gut and immune health. Front Immunol. (2021) 12:604080. 10.3389/fimmu.2021.604080 33643310 PMC7909314

[6] Bai YK Alsaidi M. Sustainable breastfeeding: a state-of-the-art review. J Hum Lact. (2024) 40(1):57–68. 10.1177/08903344231216094 38153088

[7] Smith JP Borg B Nguyen TT Iellamo A Pramono A Mathisen R. Estimating carbon and water footprints associated with commercial milk formula production and use: development and implications of the green feeding climate action tool. Front Nutr. (2024) 11:1371036. 10.3389/fnut.2024.1371036 38938671 PMC11210426

[8] World Health Organization; United Nations Children’s Fund. Global Breastfeeding Scorecard 2022: Protecting Breastfeeding through Further Investments and Policy Actions (2022). Available online at: https://www.who.int/publications/i/item/WHO-HEP-NFS-22.6 (Accessed September 12, 2025).

[9] Instituto Nacional de Estadística y Censos. Principales Resultados ENSANUT 2018. Quito: INEC (2019). Available online at: https://www.ecuadorencifras.gob.ec/documentos/web-inec/Estadisticas_Sociales/ENSANUT/ENSANUT_2018/Principales%20resultados%20ENSANUT_2018.pdf (Accessed September 12, 2025).

[10] Bandura A. Self-efficacy: toward a unifying theory of behavioral change. Psychol Rev. (1977) 84(2):191–215. 10.1037/0033-295X.84.2.191 847061

[11] Dennis CL Faux S. Development and psychometric testing of the breastfeeding self-efficacy scale. Res Nurs Health. (1999) 22(5):399–409. 10.1002/(SICI)1098-240X(199910)22:5<399::AID-NUR6>3.0.CO;2-4 10520192

[12] Dennis CL McQueen K Dol J Brown H Beck C Shorey S. Psychometrics of the breastfeeding self-efficacy scale and short form: a systematic review. BMC Public Health. (2024) 24(1):637. 10.1186/s12889-024-17805-6 38419045 PMC10903029

[13] Oliver-Roig A d’Anglade-González ML García-García B Silva-Tubio JR Richart-Martínez M Dennis CL. The Spanish version of the breastfeeding self-efficacy scale–short form: reliability and validity assessment. Int J Nurs Stud. (2012) 49(2):169–73. 10.1016/j.ijnurstu.2011.08.005 21930270

[14] Dai X Dennis CL. Translation and validation of the breastfeeding self-efficacy scale into Chinese. J Midwifery Womens Health. (2003) 48(5):350–6. 10.1016/S1526-9523(03)00283-6 14526349

[15] Pavicic Bosnjak A Rumboldt M Stanojevic M Dennis CL. Psychometric assessment of the Croatian version of the breastfeeding self-efficacy scale–short form. J Hum Lact. (2012) 28(4):565–9. 10.1177/0890334412456240 22956741

[16] Petrozzi A Gagliardi L. Breastfeeding self-efficacy scale: validation of the Italian version and correlation with breastfeeding at 3 months. J Pediatr Gastroenterol Nutr. (2016) 62(1):137–9. 10.1097/MPG.0000000000000902 26192699

[17] Gerhardsson E Nyqvist KH Mattsson E Volgsten H Hildingsson I Funkquist EL. The Swedish version of the breastfeeding self-efficacy scale–short form: reliability and validity assessment. J Hum Lact. (2014) 30(3):340–5. 10.1177/0890334414523836 24574154

[18] Amini P Omani-Samani R Sepidarkish M Almasi-Hashiani A Hosseini M Maroufizadeh S. The breastfeeding self-efficacy scale–short form (BSES-SF): a validation study in Iranian mothers. BMC Res Notes. (2019) 12(1):622. 10.1186/s13104-019-4656-7 31547846 PMC6757403

[19] Economou M Kolokotroni O Paphiti-Demetriou I Kouta C Lambrinou E Hadjigeorgiou E The association of breastfeeding self-efficacy with breastfeeding duration and exclusivity: longitudinal assessment of the predictive validity of the Greek version of the BSES-SF tool. BMC Pregnancy Childbirth. (2021) 21(1):421. 10.1186/s12884-021-03878-3 34107927 PMC8188677

[20] Maurer L Schultz A Dennis CL Alexandrowicz RW McQueen K. The breastfeeding self-efficacy scale–short form (BSES-SF): German translation and psychometric assessment. J Hum Lact. (2024) 40(3):374–85. 10.1177/08903344241254108 38831687

[21] Aluş Tokat M Okumuş H Dennis CL. Translation and psychometric assessment of the breastfeeding self-efficacy scale–short form among pregnant and postnatal women in Turkey. Midwifery. (2010) 26(1):101–8. 10.1016/j.midw.2008.04.002 18541350

[22] Chipojola R Dennis CL Kuo SY. Psychometric assessment of the breastfeeding self-efficacy scale-short form: a confirmatory factor analysis in Malawian mothers. J Hum Lact. (2023) 39(3):397–405. 10.1177/08903344221127002 36214473

[23] De Roza JG Fong MK Ang BL Sadon RB Koh EYL Teo SSH. Exclusive breastfeeding, breastfeeding self-efficacy and perception of milk supply among mothers in Singapore: a longitudinal study. Midwifery. (2019) 79:102532. 10.1016/j.midw.2019.102532 31526969

[24] Mazúchová L Maskálová E Škodová Z Koteríková D Kelčíková S Malinovská N Self-efficacy of mothers in breastfeeding and psychometric properties of the Slovak version of the BSES-SF. Kontakt. (2024) 26(1):45–51. 10.32725/kont.2024.008

[25] Basu S Garg S Sharma A Arora E Singh MM. The hindi version of the breastfeeding self-efficacy scale–short form: reliability and validity assessment. Indian J Community Med. (2020) 45(3):348–52. 10.4103/ijcm.IJCM_378_19 33354017 PMC7745807

[26] McCarter-Spaulding DE Dennis CL. Psychometric testing of the breastfeeding self-efficacy scale–short form in a sample of black women in the United States. Res Nurs Health. (2010) 33(2):111–9. 10.1002/nur.20368 20127984

[27] Trujillo A Castro-Osorio R Maldonado-Avendaño N. Traducción y validación de la escala de autoeficacia en lactancia materna BSES-SF en población colombiana. Avances Psicol Latinoam. (2024) 42(1):1–20. 10.12804/revistas.urosario.edu.co/apl/a.13013

[28] Andrade RD Bustos NC Brito CH Adasme DN López BB Parra PC Evaluación psicométrica de la escala de autoeficacia de la lactancia materna. Andes Pediatr. (2022) 93(4):470–6. 10.32641/andespediatr.v93i4.3474 37906844

[29] Juárez-Castelán MA Rojas-Russell M Serrano-Alvarado K Gómez-García J Huerta-Ibáñez A Ramírez-Aguilar M. Diseño y validación de un instrumento para medir la autoeficacia para lactar de mujeres embarazadas mexicanas. Psychologia. (2018) 12(1):25–34. 10.21500/19002386.3344

[30] Dodt RCM Ximenes LB Almeida PC Oriá MOB Dennis CL. Psychometric and maternal sociodemographic assessment of the breastfeeding self-efficacy scale–short form in a Brazilian sample. J Nurs Educ Pract. (2012) 2(3):66–73. 10.5430/jnep.v2n3p66

[31] Sandhi A Dennis CL Kuo SY. Psychometric assessment of the breastfeeding self-efficacy scale–short form: a confirmatory factor analysis of Indonesian mothers. Clin Nurs Res. (2022) 31(8):1520–8. 10.1177/10547738221112756 35904160

[32] Asgarian A Hashemi M Pournikoo M Mirazimi TS Zamanian H Amini-Tehrani M. Translation, validation, and psychometric properties of the breastfeeding self-efficacy scale–short form among Iranian women. J Hum Lact. (2020) 36(2):227–35. 10.1177/0890334419883572 31730393

[33] Yang Y Guo L Shen Z. Psychometric properties of the modified breastfeeding self-efficacy scale–short form (BSES-SF) among Chinese mothers of preterm infants. Midwifery. (2020) 91:102834. 10.1016/j.midw.2020.102834 32956984

[34] Dennis CL. The breastfeeding self-efficacy scale: psychometric assessment of the short form. J Obstet Gynecol Neonatal Nurs. (2003) 32(6):734–44. 10.1177/0884217503258459 14649593

[35] Montero I León OG. Clasificación y descripción de las metodologías de investigación en psicología. Int J Clin Health Psychol. (2002) 2(3):503–8.

[36] Kline RB. Principles and Practice of Structural Equation Modeling. 4th ed New York: Guilford Press (2016).

[37] Li CH. Confirmatory factor analysis with ordinal data: comparing robust maximum likelihood and diagonally weighted least squares. Behav Res Methods. (2016) 48(3):936–49. 10.3758/s13428-015-0619-7 26174714

[38] Lorenz T Hagitte L Prasath PR. Validation of the revised compound PsyCap scale (CPC-12R) and its measurement invariance across the US and Germany. Front Psychol. (2022) 13:1075031. 10.3389/fpsyg.2022.1075031 36619042 PMC9815509

[39] López-Guerra VM Pucha-Loarte TI Angelucci LT Torres-Carrión PV. Psychometric properties and factor structure of the satisfaction with life scale in Ecuadorian university students. Front Psychol. (2025) 16:1536973. 10.3389/fpsyg.2025.1536973 40181903 PMC11965658

[40] Beaton DE Bombardier C Guillemin F Ferraz MB. Guidelines for the process of cross-cultural adaptation of self-report measures. Spine (Phila Pa 1976). (2000) 25(24):3186–91. 10.1097/00007632-200012150-00014 11124735

[41] Bracken BA Barona A. State of the art procedures for translating, validating and using psychoeducational tests in cross-cultural assessment. Sch Psychol Int. (1991) 12(1–2):119–32. 10.1177/0143034391121010

[42] Lukat J Margraf J Lutz R van der Veld WM Becker ES. Psychometric properties of the positive mental health scale (PMH-scale). BMC Psychol. (2016) 4:8. 10.1186/s40359-016-0111-x 26865173 PMC4748628

[43] Boufellous S Sánchez-Teruel D Robles-Bello MA Lorabi S Mendoza-Bernal I. Psychometric properties of the positive mental health scale in a Spanish population. SAGE Open. (2023) 13(2):21582440231172743. 10.1177/21582440231172743

[44] World Medical Association. World medical association declaration of Helsinki: ethical principles for medical research involving human subjects. JAMA. (2013) 310(20):2191–4. 10.1001/jama.2013.281053 24141714

[45] Hambleton RK Zenisky AL. Translating and adapting tests for cross-cultural assessments. In: Matsumoto D van de Vijver FJR, editors. Cross-cultural Research Methods in Psychology. Cambridge: Cambridge University Press (2010). p. 46–70.

[46] Muñiz J Elosua P Hambleton RK. Directrices para la traducción y adaptación de los tests: segunda edición. Psicothema. (2013) 25(2):151–7. 10.7334/psicothema2013.24 23628527

[47] Flora DB Curran PJ. An empirical evaluation of alternative methods of estimation for confirmatory factor analysis with ordinal data. Psychol Methods. (2004) 9(4):466–91. 10.1037/1082-989X.9.4.466 15598100 PMC3153362

[48] Byrne BM. Structural Equation Modeling with AMOS: Basic Concepts, Applications, and Programming. 3rd ed New York: Routledge (2016).

[49] Hu L Bentler PM. Cutoff criteria for fit indexes in covariance structure analysis: conventional criteria versus new alternatives. Struct Equ Model. (1999) 6(1):1–55. 10.1080/10705519909540118

[50] Fornell C Larcker DF. Evaluating structural equation models with unobservable variables and measurement error. J Mark Res. (1981) 18(1):39–50. 10.2307/3151312

[51] Vizioli NA. Algunas consideraciones previas a la estimación de la confiabilidad de instrumentos psicométricos. Interacciones. (2021) 7:e213. 10.24016/2020.v7.213

[52] Ventura-León JL Caycho-Rodríguez T. El coeficiente omega: un método alternativo para la estimación de la confiabilidad. Rev Latinoam Cienc Soc Ninez Juv. (2017) 15(1):625–7.

[53] Gregory A Penrose K Morrison C Dennis CL MacArthur C. Psychometric properties of the breastfeeding self-efficacy scale–short form in an ethnically diverse U.K. sample. Public Health Nurs. (2008) 25(3):278–84. 10.1111/j.1525-1446.2008.00705.x 18477379

[54] Cohen J. Statistical Power Analysis for the Behavioral Sciences. 2nd ed Hillsdale (NJ): Lawrence Erlbaum Associates (1988).

[55] Sarstedt M Ringle CM Hair JF. Partial least squares structural equation modeling. In: Homburg C Klarmann M Vomberg A, editors. Handbook of Market Research. Cham: Springer (2021). p. 1–47. doi: 10.1007/978-3-319-05542-8_15-2.

[56] Balaguer-Martínez JV García-Pérez R Gallego-Iborra A Sánchez-Almeida E Sánchez-Díaz MD Ciriza-Barea E Predictive capacity for breastfeeding and determination of the best cut-off point for the breastfeeding self-efficacy scale–short form. An Pediatr (Engl Ed). (2022) 96(1):51–8. 10.1016/j.anpede.2020.12.018 34961693

